# Using Poaching Levels and Elephant Distribution to Assess the Conservation Efficacy of Private, Communal and Government Land in Northern Kenya

**DOI:** 10.1371/journal.pone.0139079

**Published:** 2015-09-25

**Authors:** Festus W. Ihwagi, Tiejun Wang, George Wittemyer, Andrew K. Skidmore, Albertus G. Toxopeus, Shadrack Ngene, Juliet King, Jeffrey Worden, Patrick Omondi, Iain Douglas-Hamilton

**Affiliations:** 1 Department of Natural Resources, Faculty of Geo-Information Science and Earth Observation (ITC), University of Twente, P.O. Box 217, 7500 AE, Enschede, The Netherlands; 2 Save the Elephants, P.O. Box 54667–00200, Nairobi, Kenya; 3 Graduate Degree Program in Ecology, Colorado State University, Ft. Collins, Colorado, 80523–1474, United States of America; 4 Kenya Wildlife Service, P.O. Box 40241–00100, Nairobi, Kenya; 5 Northern Rangelands Trust, Private Bag—60300, Isiolo, Kenya; University of Illinois at Urbana-Champaign, UNITED STATES

## Abstract

Efforts to curb elephant poaching have focused on reducing demand, confiscating ivory and boosting security patrols in elephant range. Where land is under multiple uses and ownership, determining the local poaching dynamics is important for identifying successful conservation models. Using 2,403 verified elephant, *Loxodonta africana*, mortality records collected from 2002 to 2012 and the results of aerial total counts of elephants conducted in 2002, 2008 and 2012 for the Laikipia-Samburu ecosystem of northern Kenya, we sought to determine the influence of land ownership and use on diurnal elephant distribution and on poaching levels. We show that the annual proportions of illegally killed (i.e., poached) elephants increased over the 11 years of the study, peaking at 70% of all recorded deaths in 2012. The type of land use was more strongly related to levels of poaching than was the type of ownership. Private ranches, comprising only 13% of land area, hosted almost half of the elephant population and had significantly lower levels of poaching than other land use types except for the officially designated national reserves (covering only 1.6% of elephant range in the ecosystem). Communal grazing lands hosted significantly fewer elephants than expected, but community areas set aside for wildlife demonstrated significantly higher numbers of elephants and lower illegal killing levels relative to non-designated community lands. While private lands had lower illegal killing levels than community conservancies, the success of the latter relative to other community-held lands shows the importance of this model of land use for conservation. This work highlights the relationship between illegal killing and various land ownership and use models, which can help focus anti-poaching activities.

## Introduction

Land ownership has a substantial effect on the potential use of an area for wildlife conservation [1–3], while land use also typically influences the distribution and abundance of herbivores [4–7]. In turn, animal distribution and abundance can determine the location and intensity of illegal hunting activities [8, 9]. Land under an official conservation status is traditionally associated with a higher protection and abundance of wildlife and is recognized as critical for the conservation of species [10, 11]. Nevertheless, the relationship between wildlife protection and the different ownership and land use models outside the government-protected areas has not been widely studied.

Over-hunting of wild animals is a primary driver of species decline [12, 13]. It has been designated as one of the ‘evil quartet’ drivers of extinction [14]. Through the Monitoring of Illegal Killing of Elephants (MIKE) programme of the Convention on International Trade in Endangered Species (CITES), the cause of elephant deaths is collected in selected sites across the elephant range to assess changes in illegal killing pressure over time. The monitoring data compiled under the MIKE programme across the range states provide useful information on the status of populations that have been synthesized into site, national, or continental level appraisals [15–18]. During the years 2011 and 2012, an all-time high in the poaching rate and ivory trade level was recorded across the entire African elephant range [16, 18]. A sharp increase in levels of poaching in Kenya had been reported earlier on in the year 2009 [19]. In addition to being important for assessing global trends, the MIKE data provide a unique opportunity to investigate the fine-scale spatial patterns of illegal killing at the site level.

Due to the covert nature of illegal killing and the ivory trade, it is difficult to gather information on poaching and its drivers. This is compounded by the unequal conservation effort across expansive landscapes with varied types of land ownership and land use [20]. Detailed site-level studies of elephant poaching can provide the opportunity to identify factors that contribute to rising or falling poaching levels. In Kenya, land ownership is private, communal or public [21, 22], and focused wildlife management is represented across all ownership types. Areas under distinct land use encompass varied habitat types, but their large geographical extent exceeds the spatial scale at which elephants respond to habitat heterogeneity [23]. The Laikipia-Samburu ecosystem is one of the few designated MIKE monitoring sites with a variety of land uses and ownership categories. It is home to Kenya’s second largest elephant population, estimated at approximately 6,500 elephants [24], and has been the focus of the most comprehensive carcass monitoring (yielding the largest dataset) of all MIKE sites [25]. A combination of community-based information gathering, research, and security patrols has generated a detailed dataset on elephant mortality [20].

Kenya’s national elephant management and conservation strategy underscores the need to identify land use types that are compatible with conservation [26]. Wildlife populations in the protected and unprotected areas of Kenya declined sharply from the 1980s to 2009 [27]. The general decline in migratory herbivores in Kenya is attributed to loss of dispersal areas, at least in part [28]. Despite the overall decline in wildlife numbers at the national level, the Laikipia- Samburu ecosystem has had stable or increasing numbers of some species including elephants [29]. The largest proportion of Kenya’s wildlife is found on private and communally owned land, as reflected in the Laikipia-Samburu ecosystem [27], while the combination of land ownership and land use types in this ecosystem offers an opportunity to investigate the influence of these features on poaching at the site level. This study investigated the relationships between the level of illegal killing, elephant distribution, land ownership and land uses over a period of eleven years in northern Kenya.

## Materials and Methods

Kenya Wildlife Service, the custodian of wildlife resources in Kenya, played an integral part in this study, which was thus exempt from requiring a permit.

### Study area

**Fig 1:**
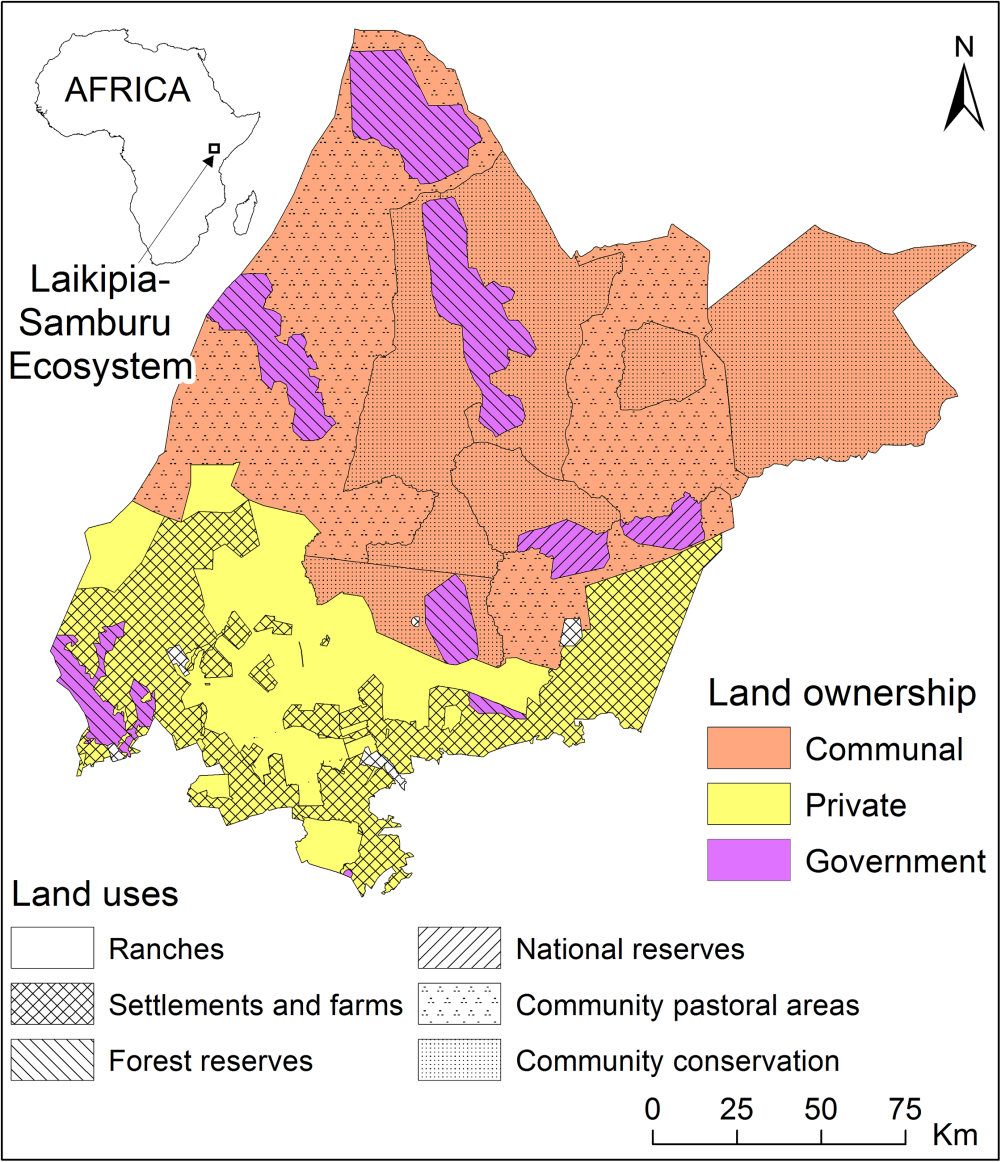
Land ownership (private, communal or government) and types of use (managed to enhance wildlife or not) in the Laikipia-Samburu ecosystem. There are three main land ownership types; private, communal and government. Ranches, community conservancies and national reserves have active wildlife protection measures in place.

The study was conducted in the Laikipia-Samburu ecosystem of northern Kenya. The ecosystem is defined by the geographic extents of the Ewaso Nyiro river and the historical elephant migration range [30]. The ecosystem lies within 0.4°S to 2°N, 36.2°E to 38.3°E, and encompasses an area of 33,817 km^2^ [31]. A wide range of habitats are linked with the elevation and climatic gradients that characterize the region: from cool, wet highlands in the south to hot, dry lowlands in the north [30]. Rugged mountains interrupt the otherwise gently undulating open landscape, which elephants would generally avoid [32]. The confirmed Laikipia-Samburu elephant range encompasses six major land use types: community conservancies, private ranches, communal pastoral areas, state-protected forest reserves, settlements mainly under sedentary subsistence production, and the national reserves (Fig 1).

**Fig 2:**
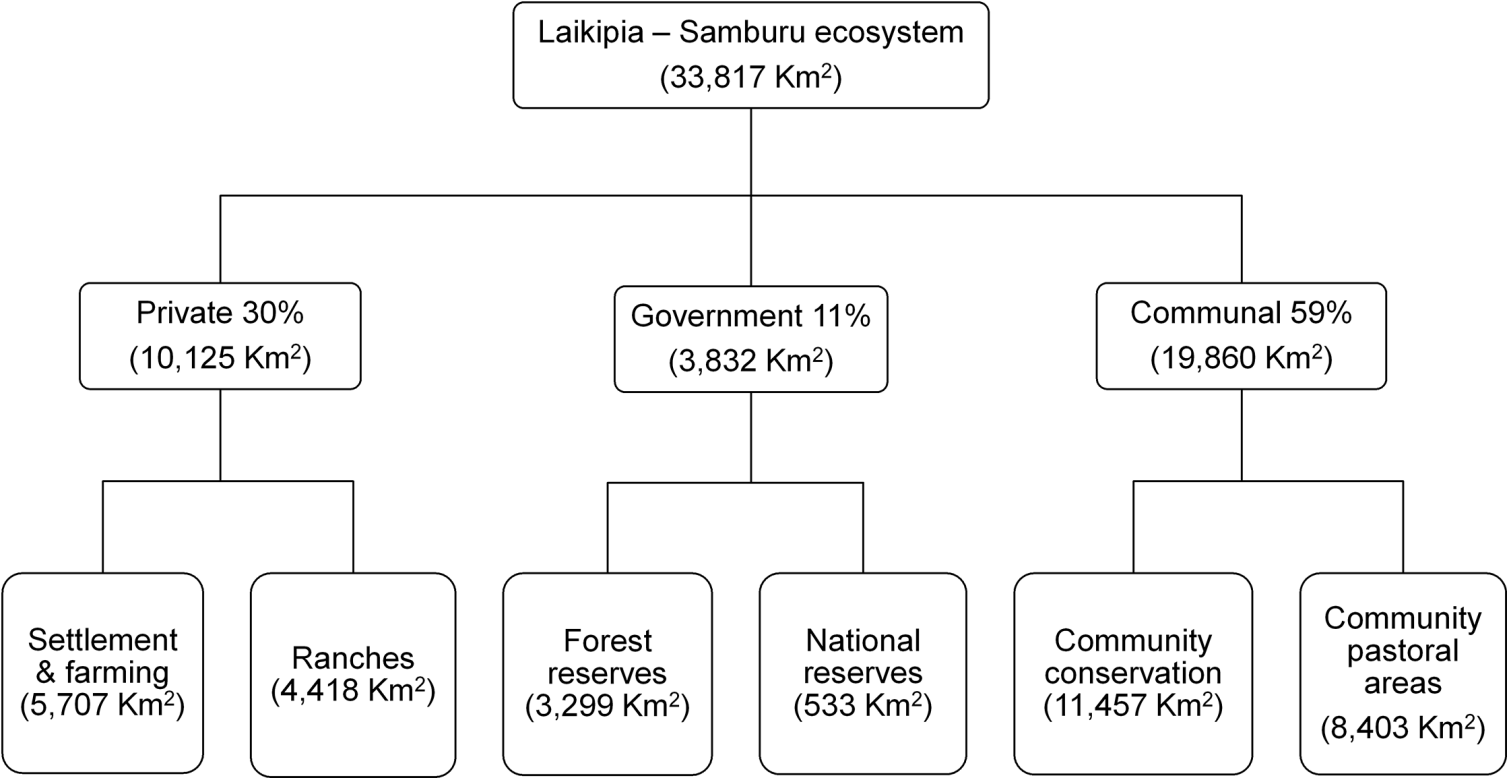
Land ownership and the corresponding land use types in the Laikipia-Samburu ecosystem.

The private, government and community lands comprise 30%, 11% and 59% of the landscape, respectively. The area of land under each different land use type ranges from 533 km^2^ to 11,457 km^2^ (Fig 2). Non-conserved communal land is occupied by nomadic pastoral communities, and inhabited by both livestock and wildlife, but it lacks any systematic security patrolling. There are also communities that actively manage their land for wildlife protection (i.e., community conservancies), and have trained (and in some cases armed) rangers to patrol the conservancies. The government land comprises national reserves managed for wildlife conservation, and forest reserves, which are national heritage sites but with no active management for wildlife. There are three national reserves in the ecosystem, Samburu, Buffalo Springs and Shaba. These are located at the centre of the ecosystem, but are relatively small (533 km^2^ in total), representing only 1.5% of land under the confirmed elephant range. The national reserves are managed by local government authorities, which employ armed rangers to safeguard wildlife. Unauthorized access in national reserves is prohibited, although there are concessions for communal use and access by surrounding and/or nomadic communities is common but regulated. The forest reserves are managed by the national government and they often coincide with mountain ranges. Unlike the national reserves, the communities living around forests have uncontrolled access to them. They use the forests as additional grazing land. The southern limit of the Laikipia-Samburu ecosystem is primarily private land (i.e., settlements and ranches). In the settlements, the land is highly subdivided into plots of less than ten hectares. A few of these plots are not yet permanently occupied, but are instead utilized as extra grazing areas by neighbours. Over 50 private ranch properties, ranging from approximately 10 hectares to over 35,000 hectares, are managed for commercial cattle production, with owners generally allowing wildlife access on their properties. Some of the ranches have tourism establishments and activities. They have establishments such as hotels, lodges and campsites, etc., whereas activities include day-trippers/day safaris and tour operator visits.

### Total aerial count of elephants

To assess elephant distribution, population status and trends, total aerial counts were conducted in June 2002 (dry season), November 2008 (wet season) and November 2012 (wet season) using standard total aerial counting techniques [33, 34]. High-wing Cessna aircraft (10 in 2002, 10 in 2008 and 13 in 2012) were used in each of the week-long counting exercises. The interval between the flight lines was set at one or two kilometres, depending on visibility, to ensure all the ground was scanned and all the elephants were counted. The waypoints and corresponding elephants counts were assigned to land ownership and land uses for further analyses. The average densities of elephants were estimated from the three counts yielding a relative abundance across the wet and dry seasons.

### Collecting elephant mortality data

Information on incidences of elephant mortality was gathered through a network of nomadic herders, researchers, community conservancy scouts, private ranch managers, and Kenya Wildlife Service rangers [20]. The information from herders and ranch managers was verified by a field visit to the carcass by a Kenya Wildlife Service ranger, a trained community scout, or a researcher. A standard data sheet devised by the MIKE Technical Advisory Group was completed for each carcass, including the estimated date of death, GPS coordinates and the cause of death [35]. Four causes of death were recognized, i.e., poached, human-elephant conflict, problem animal control (killed by authorized personnel in defence of life or property), and natural mortality. Where it was not possible to identify the cause of death with certainty, the cause of death was listed as ‘unknown’. A total of 2,403 dead elephants were recorded from 2002 to 2012. The breakdown of number of carcasses from across the land use types is provided as Table 1.

**Table 1:** The number of elephant carcasses recorded from 2002 to 2012, their cause of mortality and the average number of live elephants recorded within different land use types in the Laikipia-Samburu ecosystem. Notes: HEC refers to elephant mortality resulting from human elephant conflict incidences. PAC refers to problem animal control, i.e., elephant mortality as a result of killing of problematic elephants by authorised personnel. The proportionate cause of mortality within each land use type is indicated in brackets. The live elephants refers to the average number recorded within land under each use type in the years 2002, 2008 and 2012.

Land use	Area (km^2^)	Live elephants	Causes of elephant mortality				
			HEC	Natural	PAC	Poached	Unknown
Settlement and farming	5,707	73	14(12%)	29(25%)	27(23%)	30(26%)	16(14%)
Ranches	4,418	2652	43(7%)	235(37%)	39(6%)	220(34%)	103(16%)
Forest reserves	3,299	407	55(14%)	95(25%)	13(3%)	154(40%)	64(17%)
National reserves	533	602	2(1%)	80(56%)	2(1%)	41(28%)	19(13%)
Community conservation	11,457	1872	82(10%)	259(33%)	8(1%)	308(39%)	139(17%)
Community pastoralism	8,403	785	41(13%)	84(26%)	6(2%)	125(38%)	70(21%)

Search efforts by herders and patrol officers on ranches and in pastoral areas were not recorded. The search effort was generally expected to vary between the different land use types, but constant within each land use type over time. Likewise, the financial and human resources deployed by land managers were not available. Preliminary analyses of the effectiveness of the data collection protocol were performed using data for the first three years, 2001 to 2003, and showed that the numbers of carcasses due to various causes did not vary considerably between the different participants in the data collection network [20]. The Proportion of Illegally Killed Elephants (PIKE) has been validated as a reliable measure of the severity of illegal killing in monitoring sites, irrespective of the availability of effort information [20, 25, 36]. The PIKE is calculated as:

$$\text{PIKE}\;\left(\%\right)=\frac{Number\;of\;illegaly\;killed\;elephants}{Total\;number\;of\;dead\;elephants\;recorded}\;\times 100$$ (1)

PIKE values exceeding 54% have been identified as indicative of declining populations [16, 18]. The ratio of dead to all the counted live and dead elephants, i.e., the carcass ratio, provides insight into population trends [20, 37], and was examined alongside the carcass monitoring data. This study used the ground-based carcass count together with the aerial live-elephant count to determine the carcass ratio.

### Statistical analyses

The observed distribution of elephants per land use category was compared to the expected distribution using a Chi-square test. The expected distribution was derived from a null or random distribution assumption (the study area’s average elephant density multiplied by the area of land use zone). Spatial and temporal variation in the level of poaching over the 11-year study period were analysed using a logistic regression generalized linear model (GLM) (binomial family with a logit function and implemented with the “lme4” package in R) [38]. The response variable was the number of elephant carcasses found as a binary outcome of two main causes of deaths, i.e., illegally killed or not illegally killed. The probability of illegal killing of elephants was modelled using a bivariate covariate for each of the land ownership types (private, communal or government). Land use type, either managed for wildlife or not, was also assigned a bivariate covariate. Elephant density was factored in the model as a continuous variable. Time was factored in as “year of death”. The land use type officially designated for wildlife conservation (national reserves) was used as the reference covariate. Models with different combinations of covariates and their interactions were fitted and compared using the second-order Akaike Information Criterion (AICc) [39].

Some of the community conservancies were established more recently than others. Their development differs in terms of staff recruitment and conservation budgets, but there were no comparable management records available for all the conservancies to enable us to perform a systematic analysis of these factors. To limit the impact of such variability, only the fully operational conservancies as of 2005 were ascribed such status in our analysis. Those not fully established were lumped together with communal grazing areas.

Upon breaking down the dataset into individual land use types by year there were wide variations in sample sizes. Consequently, the annual PIKE values across the individual land use and ownership types were not normally distributed. Due to these irregularities, non-parametric tests were applied to assess differences in PIKE across land use types. The differences in PIKE levels were compared among the land uses under the same ownership category using the Kruskal-Wallis test. The differences in PIKE across the six land use types were tested using pairwise Mann-Whitney tests. Pearson’s product-moment correlation coefficient (*r*) was used to assess the correlation between the study area’s carcass ratios and PIKE within the land use types. Pearson’s *r* was also used to test for the relationship between the number of live elephants and the number killed illegally, as well as the number of deaths from natural causes. A linear regression was used to test the significance of the trend in PIKE level from 2002 to 2012. All tests of statistical significance were conducted at α = 0.05.

## Results

### Distribution of elephants in relation to land ownership and land uses

**Fig 3:**
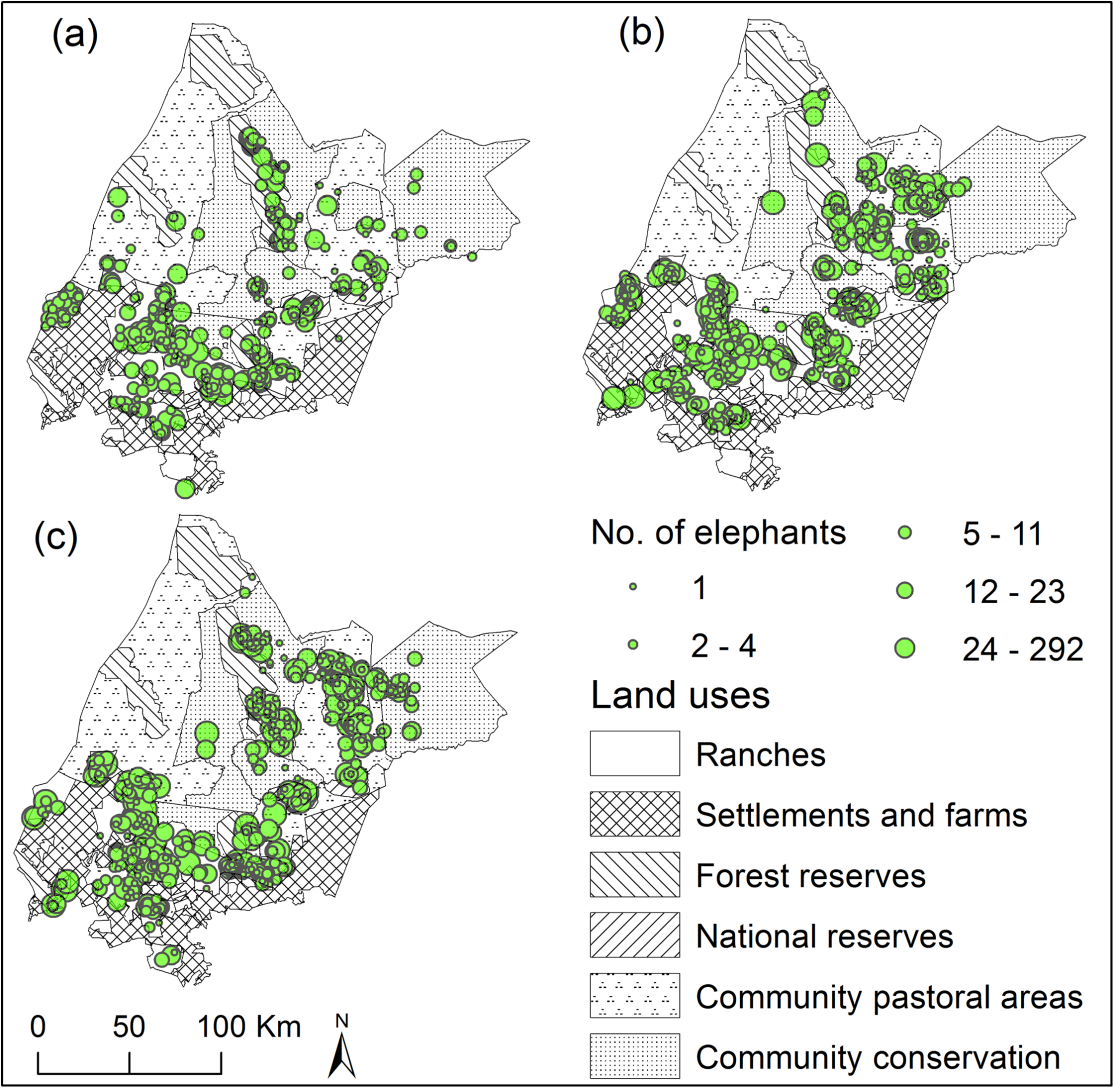
The distribution of elephants in the Laikipia-Samburu ecosystem derived from total aerial counts in (a) 2002 (n = 5,447), (b) 2008 (n = 7,415), and (c) 2012 (n = 6,365). Elephants are found in large numbers within private ranches and the national reserves.

A total of 5,447 elephants were counted in 2002, 7,415 in 2008 and 6,365 in 2012 (Fig 3). There were significant differences between the observed and expected numbers (based on land area) of live elephants across the three land ownership types (χ^2^ = 776.6, *P* < 0.001) and also within the six land uses (χ^2^ = 301.7, *P* < 0.001). The site’s average elephant density was 0.314 elephants per square kilometre. The private ranches and national reserves were higher than the average at 0.537 and 0.993 elephants per square kilometre, respectively. There was a close match between the observed and expected number of elephants within the community conservancy areas (conservancies comprise 33.9% of the elephant range and hosted 29.3% of the elephants). The communal land under pastoralism, comprising 24.8% of the elephant range, hosted half of the expected number of animals at only 12.3% of the elephant population.

### PIKE on land under different ownership and uses

**Fig 4:**
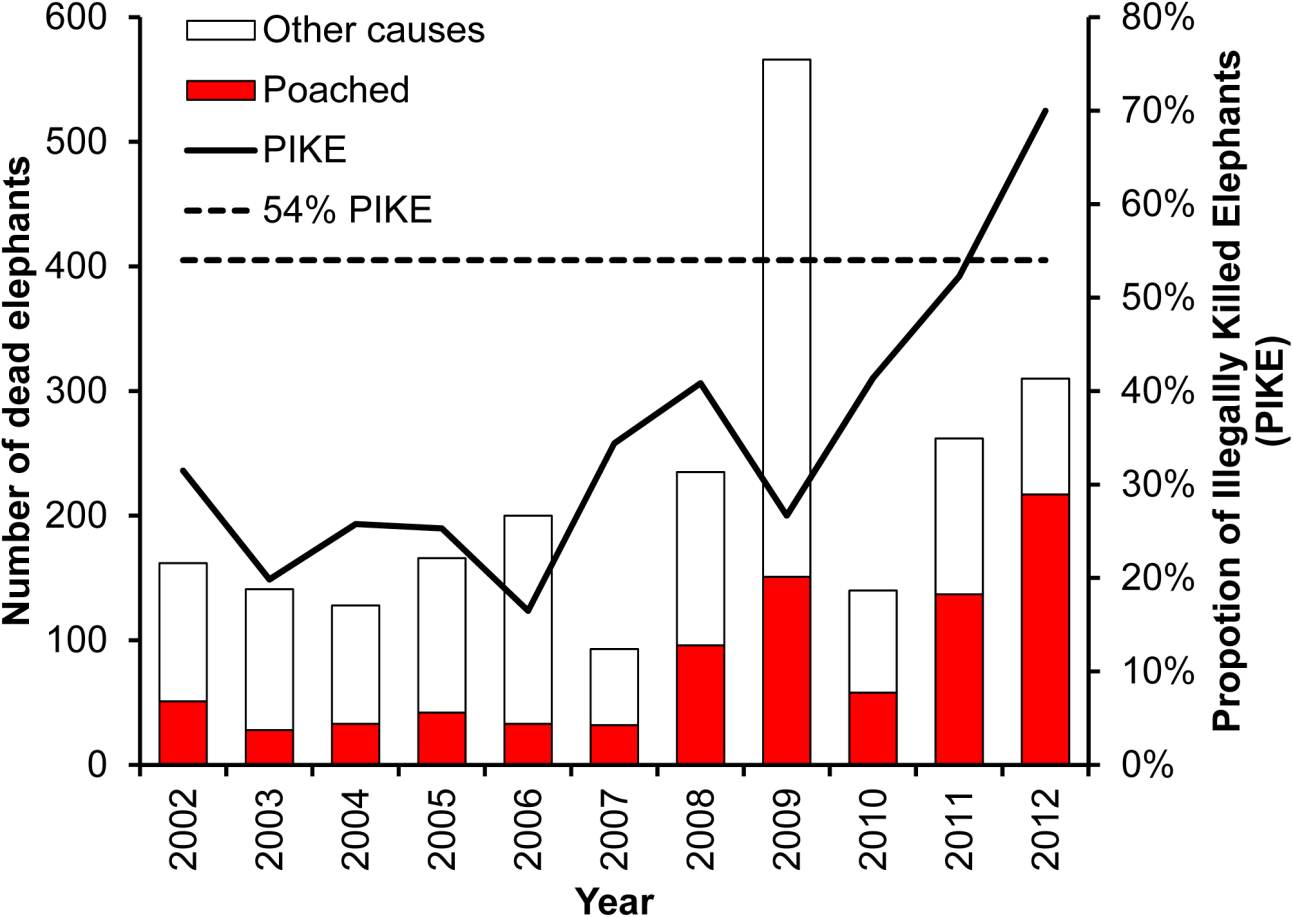
The numbers of elephants that died from poaching and other causes from 2002–2012. The dotted line indicates the level of poaching (i.e., 54% PIKE) beyond which populations cannot compensate via births and decline is imminent.

**Fig 5:**
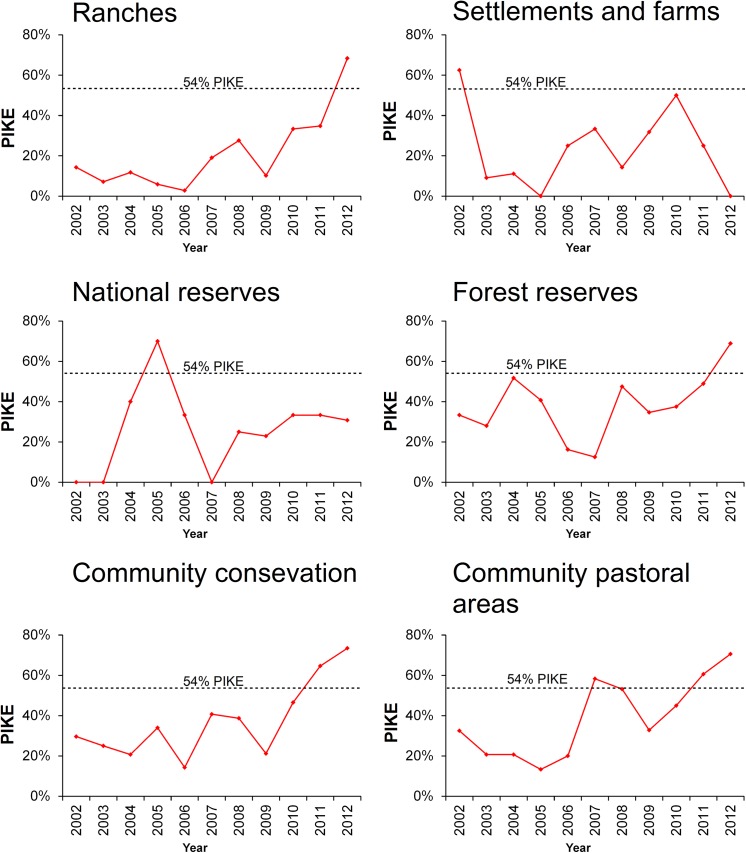
Trends in the level of proportion of illegally killed elephants (PIKE) across the different types of land use for 2002–2012. An increase in PIKE from 2010–2012 was recorded in most of the land use types.

The overall PIKE increased significantly over the 11 years of the study (*R* ^2^ = 0.8, n = 10, *P* < 0.05)(Fig 4). The private ranches, settlements and national reserves had the lowest levels of average annual PIKE for the entire study period at 21%, 24% and 26% respectively. On the other hand, community conservation areas, forest reserves, and community pastoral areas had higher levels of average annual PIKE at 37%, 38% and 39% respectively. Annual PIKE increased in each land use category except for the national reserves and settlement areas (Fig 5).

**Fig 6:**
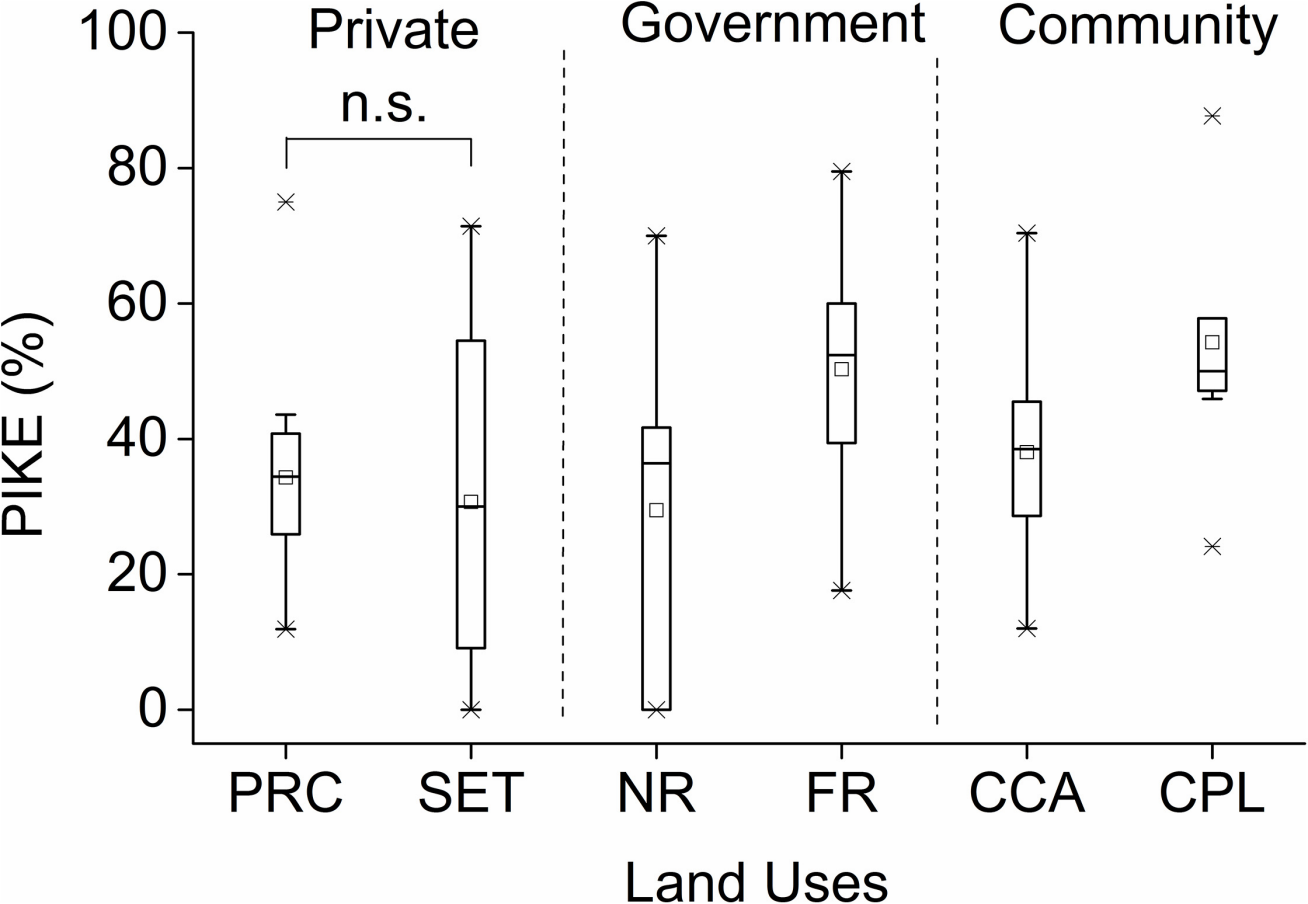
The proportion of illegally killed elephants (PIKE) in the six land use types. Land uses are abbreviated as PRC (private ranches and conservancies), SET (settlements and farms under private ownership), NR (national reserves), FR (forest reserves on government-owned land), CCA (community conservation), and CPL (community pastoralism on communally owned land). There was no significant difference (abbreviated as ‘n. s.’ on the figure) in the PIKE level between private ranches and settlements Mann-Whitney U test (*P* = 0.96).

The PIKE levels did not differ significantly between the three different ownership types if land use within each type was not accounted for (Kruskal-Wallis χ^2^ = 5.248, *P* = 0.073). There were significantly lower levels of PIKE in areas managed for wildlife on government land (i.e. national reserves had a lower PIKE than forest reserves) (Mann-Whitney test: *U* = 19.682, *Z* = 2.405, *P* = 0.016), as well as lower levels in conservancies relative to pastoral areas within community land (Mann-Whitney test: *U* = -16.182, *P* = 0.048). However, there was no difference in PIKE found between private ranches and settlements (Mann-Whitney test: *U* = 0.409, *Z* = 0.05, *P* = 0.96) (Fig 6).

A set of eleven generalized linear models with different combinations of covariates were constructed (Table 2). The top two models were selected using the second-order AICc (Table 3). The coefficients of the top model are shown in Table 4. The top model featuring only land use, its ownership type and time factor (i.e., year of observation) explain 38% of the variation seen in the level of illegal killing of elephants in the Laikipia-Samburu ecosystem (Table 5).

**Table 2:** Candidate models in the analyses of the relationship between the probability of illegal killing of elephants (*P*_illegal_), land ownership, land use and elephant densities. ‘WF’ denotes wildlife-friendly land regardless of ownership. The asterisk between covariates shows the only interactive effects of ownership and use that were found to be significant predictors of illegal killing.

Model	Model description
1	*P*_illegal_ = β_0_ + β_1_(year) + β_2_(private) + β_3_(community) + β_4_(WF) + β_5_(private*WF)
2	*P*_illegal_ = β_0_ + β_1_(year) + β_2_(density)+ β_3_(private) + β_4_(community) + β_5_(WF)+ β6(private*WF)
3	*P*_illegal_ = β_0_ + β_1_(year) + β_2_(WF)
4	*P*_illegal_ = β_0_ + β_1_(year) + β_2_(WF)+ β_3_(density) + β_4_(community)
5	*P*_illegal_ = β_0_ + β_1_(year) + β_2_(WF)+ β_3_(density) + β_4_(private)+ β_5_(community)
6	*P*_illegal_ = β_0_ + β_1_(year) + β_2_(WF)+ β_3_(private) + β_4_(community)
7	*P*_illegal_ = β_0_ + β_1_(year) + β_2_(WF)+ β_3_(density)
8	*P*_illegal_ = β_0_ + β_1_(year) + β_2_(WF)+ β_3_(density) + β_4_(private)
9	*P*_illegal_ = β_0_ + β_1_(year) + β_2_(density)
10	*P*_illegal_ = β_0_ + β_1_(private) + β_2_(community) + β_3_(WF) + β_4_(private*WF)
11	*P*_illegal_ = β_0_ + β_1_(density) + β_2_(private) + β_3_(community) + β_4_(WF)+ β_5_(private*WF)

**Table 3:** Selection statistics for the top two models of the analyses of relationships between the probability of illegal killing of elephants, land ownership, land uses and elephant density. The coefficient for each variable is presented alongside each variable. ‘WF’ denotes wildlife friendly land regardless of ownership.

Model	AICca	Δib	Wic
-290.15 + 0.15 (year) -0.71(private) + 0.24 (community) -0.89(WF) +1.16 (Private*WF)	465.7	0.00	0.76
-289.81 + 0.15(year) +0.18(density) -0.67(private) + 0.29(community) -0.94(private*WF)	468.0	2.28	0.24

* denotes interactive effects. aAICc: Second-order Akaike Information Criterion; bΔi: delta AIC values; cwi: Akaike weights.

**Table 4:** The coefficients of the covariates of the top model and their statistical significance.

	Estimates	Standard error	Test statistic (Z)	Significance (P)
Intercept	-290.147	33.727	-8.603	< 0.001
Year	0.145	0.017	8.604	< 0.001
Private land	-0.714	0.243	-2.934	0.003
Communal land	0.243	0.124	1.966	0.049
Managed for wildlife	-0.886	0.119	-7.472	< 0.001
Private*managed for wildlife	1.159	0.266	4.364	< 0.001

* denotes interactive effects

**Table 5:** The deviance explained by various covariates of the top model for the probability of illegal killing of elephants in the Laikipia-Samburu ecosystem. Land use and time factor explain 38% of the variation in illegal killing.

	Deviance	Residual deviance	Deviance explained
NULL	392.03		
Year	80.52	311.51	20.54%
Private land	8.56	302.95	22.72%
Communal land	0.42	302.54	22.83%
Wildlife friendly use	39.06	263.48	32.79%
Private*Wildlife friendly use	19.98	243.51	37.88%

* denotes interactive effects

Aerial survey results found that the study area had an average carcass ratio of 3.5. The numbers of carcasses from natural mortality in the different land use categories were significantly correlated with the numbers of live elephants (Pearson’s *r* = 0.951, *P* = 0.004). In contrast, the numbers of carcasses from poaching were not correlated with the number of live elephants (Pearson’s r = 0.205, *P* = 0.696). The average carcass ratios in the entire study area for the three census years were significantly correlated with the corresponding proportions of poached carcasses (Pearson’s *r* = 0.997, *P* = 0.003), but not with the proportion of natural mortalities (Pearson’s *r* = -0.906, *P* = 0.094).

## Discussion

### Elephant distribution, land ownership and land use

The lands managed by private ranches and community conservancies are manifestly important for conservation because they have a much higher number of elephants on them than we had expected to find. Elephants move from the private ranches to the settlement areas under the cover of darkness, especially during the crop-growing seasons [40]; this behaviour may lead to their occupancy of the settlements being under-represented by aerial counts, which are conducted during daylight hours. This nocturnal behaviour has been reported in the southern part of the Laikipia-Samburu ecosystem where private ranches border dense and permanent settlements [40]. Unlike in the settlements and ranches interface, the diurnal movement of elephants between pastoral community land and the protected areas is minimal [41]. We found the community conservancies are important for the conservation of elephants because they have significantly higher elephant densities relative to the unprotected pastoral areas. The community lands are also important for connectivity in the greater ecosystem [42]. However, wildlife access to prime grazing areas of communal land is, at times, affected by conflicts amongst pastoral tribes seeking control of such areas. A key consequence of establishing conservancies has been the peaceful resolution of disputes and promotion of harmonious co-existence [43], which has benefited both wildlife and people. In the Samburu-Laikipia ecosystem, armed conflicts were leading to incursions into the prime wildlife habitats, including the national reserves. These were causing the wildlife to disperse elsewhere. The occupation of protected areas by illegally armed nomadic pastoralists during bouts of tribal conflict, for example in Shaba National Reserve in the year 2010, further hinders the security patrol efforts and puts elephants and other wildlife at greater risk of poaching.

### Temporal trend in poaching

Analysing the site level dynamics of poaching in landscapes under varied ownership and uses can inform management on where to focus anti-poaching activities. The increase in poaching over time in the Laikipia-Samburu ecosystem was consistent with the internationally observed trend of a general increase in the illegal killing of elephants across the African elephant range [16]. It likely reflects the increasing black market price of ivory in the region and the increasing trafficking of illegal ivory through Kenya during this period [18]. The temporal change in levels of poaching also interact with land use categories (see discussion below). In the year 2010, the private ranches that had previously sustained relatively low levels of poaching experienced more poaching as well.

In 2009, there was a severe drought that led to the death of an unusually high number of elephants [44]. The number of carcasses recorded in the drought year reached an all-time high of 566, compared to an average of 160 carcasses per year in the preceding years. In this drought year, 286 deaths were confirmed to have been from natural mortality. The drought-related natural deaths led to a marked reduction of PIKE for the year. Nevertheless, the absolute numbers of poached elephants increased from 96 in 2008 to 151 in 2009. Since we were unable to control for security patrol efforts, we cannot infer poaching trends from the absolute numbers of carcasses [20, 36]. However, other approaches relying on intensive monitoring of individual elephants captured an increase in poaching rates in 2009 [18].

Poached elephant carcasses found in the national reserves were mainly of elephants shot outside the reserves, but which succumbed in the reserves as they sought refuge [44]. A number of injured elephants were also seen in the national reserves and treated for gunshot wounds. Consistent with the observed increase in poaching levels throughout Africa, the proportion of poached elephants in forest reserves rose steadily from the year 2010 to an all-time high of 76% in 2012; this was higher than in any other land use type. The unhindered access to the forest reserves may make it easier for the poachers to operate.

The private ranches host approximately 42% of elephants in the ecosystem and had a low level of PIKE relative to all the land uses until the poaching surge in 2010–2012. In 2012, the PIKE went up to 77%. An average of 58 dead elephants (from various causes) was recorded each year on private ranch land. Though we did not analyse PIKE within individual ranches due to small sample sizes, we observed that the surge in poaching did overwhelm a few of the ranches (Ol ari Nyiro, ADC Mutara and Ngorare ranches) that suffered unauthorized incursions by pastoralists. The leading causes of mortality in the settlements were problem animal control and human elephant conflict. To understand why PIKE levels were not different between the two management levels on private lands (i.e., ranches and settlements), we need to have finer scale metrics including individual land owner’s investments towards elephant protection, which however are not available. However, we suspect that there is minimal disparity in the level of security investments and other infrastructural developments across the privately owned lands. The community pastoral areas had the highest overall levels of poaching during the entire study period (average annual PIKE = 49.8%), but the PIKE in these areas also increased in 2010–2012 in line with the trend seen in the other land use types. Likewise, the community conservancies had lower levels of poaching until the year 2010 when PIKE started increasing. Overall, it is apparent that there was a major change in illegal killing activity during 2011 and 2012, when even better protected areas experienced markedly higher levels of poaching.

Variation in carcass ratio can be attributed to sampling effects as well as demographic drivers of immigration, emigration, births and deaths [37]. The positive correlation between PIKE and carcass ratio is an indication that the variation in carcass ratio can, at least in part, be attributed to poaching. The 14.2% decline in elephant numbers in the years 2008–2012 (from 7,415 to 6,365) can most likely be attributed to the drought and to poaching, rather than migration as the counts were conducted at exactly the same time of the year (season) and the dispersal areas were limited. A lack of correlation between PIKE and local elephant densities shows that the activity of poachers was not influenced by the local elephant densities. The conservation efforts in various land use units are the most likely determinants of where and when poachers strike, since we have shown that land use and time explains 38% of the variation seen in illegal killing of elephants in this study. Encouraging and promoting land owners to adopt land use types that recognize the importance of protecting wildlife would thus substantially reduce poaching levels. The rest of the variation in poaching levels could be explained by other factors related to human activities or variation in law enforcement which we had no data for, and also possibly by natural resource distribution which was beyond the scope of this study.

### Poaching, land use and land ownership

Non-protected elephant habitats are important to the conservation of elephants [16], but are often the areas mostly under threat [45, 46]. In the Samburu-Laikipia ecosystem, which is largely unprotected, we found poaching levels were not simply a function of elephant density (the primary correlate of natural mortality in the system). This differs from a parallel analysis conducted in a protected area, where poachers were selecting sites based on elephant population density [9]. Rather, here our results show that land use has a strong influence on the level of poaching in the Laikipia-Samburu system, but this relationship cannot be predicted by ownership type alone. Instead, we found that specific types of land use within ownership categories were more clearly related to levels of poaching.

Land outside the protected areas is pivotal for elephant conservation in the Laikipia-Samburu ecosystem because it accounts for 98.5% of the elephant range. The unprotected land under private ranching and community conservation had the highest densities of elephants, indicating their importance for elephant conservation in the ecosystem. Significantly higher densities of elephants in the community conservancies than in the community pastoral areas indicate the success of this model of conservation: management of wildlife alongside communal grazing. Despite lower densities of live elephants and higher ratios of illegally killed carcasses, the unprotected community pastoral land is important for connecting the formally protected areas and the wildlife friendly private ranches and conservancies in the greater ecosystem [47].

## Conclusions and Recommendations

Levels of poaching in the Laikipia-Samburu ecosystem are heterogeneous in space and time and strongly related to land use type (more than to ownership model). The most successful models of conservation (land uses), based on elephant density and levels of illegal killing, were private ranching and community conservation. This study suggests that how local interventions to reduce elephant poaching can be more effective if they are focused on the most affected areas, and not necessarily on where elephants densities are highest (although both are important).

Our results indicate that the promotion of ecotourism and related facility development in communal areas has translated into better protection for elephants. In addition, ecotourism is recognized as a key contributor to the economy of the private ranches [48, 49]. In the community pastoral land and forest reserves, where poaching incidences were remarkably high, enhancing security patrols is an important measure. The community land has the highest potential for elephant conservation. Enhancing incentives for wildlife conservation in these pastoral communities could be beneficial to wildlife conservation in the ecosystem. Financial investments in anti-poaching and elephant protection should prioritize the newly established conservancies to accelerate their growth towards self-sustainability. A further study on the drivers (specific human activities and environmental factors) of poaching which transcend land use delineations is recommended.
